# Effect of Microwave Disinfection on Compressive and Tensile Strengths of Dental Stones

**DOI:** 10.5681/joddd.2013.007

**Published:** 2013-02-21

**Authors:** Mahmood Robati Anaraki, Elnaz Moslehifard, Soran Aminifar, Hamed Ghanati

**Affiliations:** ^1^Dental and Periodontal Research Center, Tabriz University of Medical Sciences, Tabriz, Iran; ^2^Assistant Professor, Department of Prosthodontics, Faculty of Dentistry, Tabriz University of Medical Sciences, Tabriz, Iran; ^3^Assistant Professor, Department of Prosthodontics, Faculty of Dentistry, Kurdistan University of Medical Sciences, Sanandaj, Iran; ^4^Undergraduate Student, Faculty of Dentistry, Tabriz University of Medical Sciences, Tabriz, Iran

**Keywords:** Compressive strength, dental stone casts, microwave disinfection, tensile strength

## Abstract

**Background and aims:**

Although microwave irradiation has been used for disinfection of dental stone casts, there are concerns regarding mechanical damage to casts during the process. The aim of this study was to evaluate the effect of microwave irradiation on the compressive strength (CS) and diametral tensile strength (DTS) of stone casts.

**Materials and methods:**

In this in vitro study, 80 cylindrical type III and IV stone models (20 × 40 mm) were prepared and divided into 8 groups of 10. The DTS and CS of the specimens were measured by a mechanical testing machine at a crosshead speed of 0.5 cm/min after 7 times of frequent wetting, irradiating at an energy level of 600 W for 3 minutes and cooling. Data were analyzed by Student’s t-test.

**Results:**

Microwave irradiation significantly increased DTS of type III and IV to 5.23 ± 0.64 and 8.17 ± 0.94, respectively (P < 0.01).

**Conclusion:**

According to the results, microwave disinfection increases DTS of type III and IV stone casts without any effects on their CS.

## Introduction

Gypsum casts may cause cross-infection between the clinic and the laboratory during prosthodontic treatments.^1-3^ Many techniques have been recommended for disinfection of casts and elimination of cross-contamination cycle, with almost all being chemical methods. Despite being effective procedures for disinfection, the materials and the processes involved have been shown to exert detrimental effects on the physical and mechanical characteristics of dental casts.^4-6^ In addition, further precautions restricting the use of these materials include their toxicity to humans and the environment, generation of resistant species, and complexity and sensitivity of the techniques.^7-10^

To overcome these restrictions, other methods such as microwave disinfection of acrylic resins and stone casts have been proposed and assumed effective.^11-15^ However, being a new method, there are not sufficient data on the effect of microwave irradiation on the physical, including surface roughness and dimensional accuracy, and mechanical characteristics of stone casts. The mechanical properties including compressive strength (CS) and diametral tensile strength (DTS) in this regard have, in particular, direct influences on fracture and abrasion resistance of dental stone casts. However, previous studies on the subject have yielded controversial results.^16-18^ Moreover, these studies have not investigated the effect of repeated irradiations, as considered in chemical disinfection studies.^19,6^

In spite of the controversy, many studies emphasize that only low-to-medium levels of energy are safe and preserve the CS, DTS and dimensional accuracy of stone casts.^20-22^ One study has shown that the sterilization of stone casts can be achieved at 600 W in 3 minutes,^23^ an energy level which is in the safe range for stone casts. Contrary to other studies which have evaluated the effects of irradiation on mechanical properties of stone casts in one irradiation cycle, if according to the conditions of this study, it could be shown in multiple disinfection procedures by irradiation, similar to the condition of casts in the clinic and laboratory, that the DTS and CS of stone casts do not decrease, this technique can be applied as a simple and reliable disinfection technique, without the disadvantages of chemical techniques, in every stage of clinical procedures.

The hypothesis tested in this study was that DTS and CS of stone specimens are not affected by microwave irradiation at 600 W in seven separate exposures for three minutes in each run in comparison to the control group.

## Materials and Methods

### Preparation of Specimens

**Figure 1:**
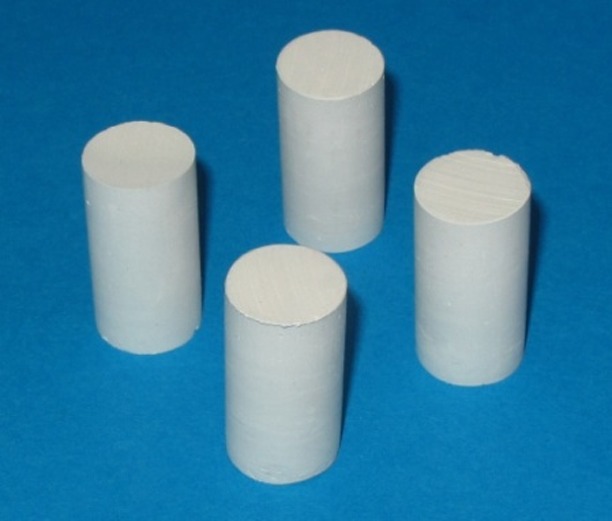
Cylindrical stone samples (20 mm × 40 mm) prepared for testing.

Type III (Elite Model, Zhermack, Italy) and type IV (Elite Base, Zhermack, Italy) dental stones were used for the purpose of this in vitro study. The water/powder ratio used was according to manufacturer’s instructions. A silicon rubber mold (Terma Lab, Major, Italy) with four cylindrical spaces, 20 millimeter in diameter and 40 millimeter in height, were prepared from stainless steel models according to ANSI/ADA No. 25.^22^ A weighed amount of powder was added to water in a rubber bowl and mixed manually to a smooth consistency and then placed in a vacuum mixer (Degussa, Frankfurt, Germany) for 25 seconds under the vacuum condition of 0.8 to 1 mm Hg to eliminate porosity; then the mixture was gently poured into the silicon mold on a vibrator. Subsequently, a glass plate was placed on the silicon mold to create a smooth and parallel surface. Forty-five minutes after mixing, the cylindrical specimens were removed from the silicon mold and weighed on a digital weighing machine. Based on similar studies,^19,6^ ten specimens were prepared for each study group; on the whole, 80 specimens were prepared with the same dimension and weight without any obvious defects at ×3 magnification in this manner and stored at ambient temperature of 23±2°C and a relative humidity of 45–50% (Figure 1).

### Irradiation and Test of Strength

Twenty-four hours after preparing the stone specimens, half of them were randomly separated for irradiation. In the first stage, the casts were wetted by spraying water until they did not absorb any more, but without any visible water on surfaces when they were placed in the microwave oven to prevent micro-explosion and cracking following the rapid formation and release of vapor.

The specimens were irradiated in a microwave oven (Samsung, PG 3210, 1450 MHz, China) for 3 minutes at 600 W. To prevent heat shock, the casts were allowed to cool in air. The sequences of wetting, irradiation and cooling were repeated seven times, while a container with 400 mL of water was placed in the chamber to protect the magnetron of the microwave oven from excess heat after all the moisture of the specimens evaporated.

**Figure 2:**
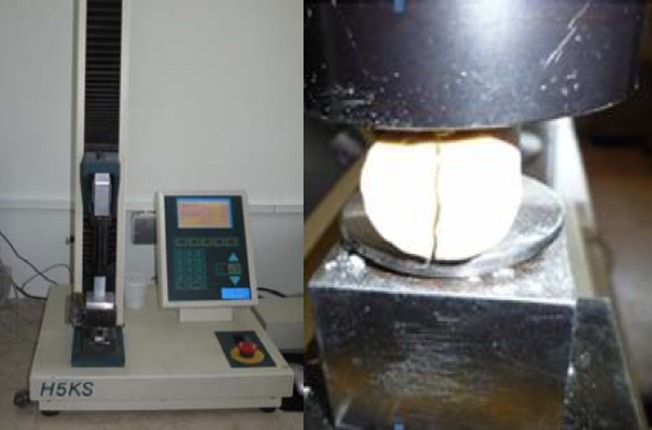
Mechanical testing machine (A). Measuring the tensile strength of the stone model (B).

One week after the production of casts, a mechanical testing machine (H5KS, Instron, Shimatzu, Japan) was used for testing the irradiated and un-irradiated specimens with a load of 10 Kg at a crosshead speed of 0.5 cm/min (Figure 2A).

To evaluate the CS of the specimens, each sample was placed in a perpendicular position in the testing machine and the maximum force (MPa) for breaking was recorded for CS.

To measure the DTS, each specimen was placed diagonally in the testing machine and after measuring the maximum force for breaking the specimen (Figure 2B), the following formula was used to calculate DTS:

**Figure:**
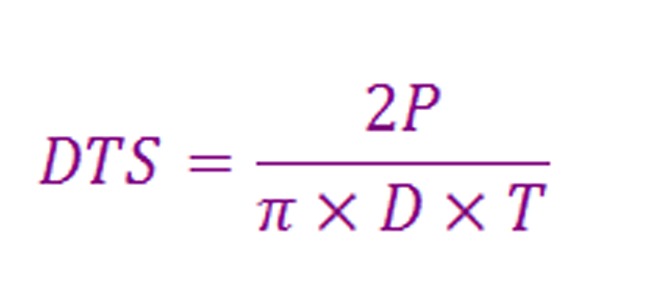


where “P” is the force applied, “D” is the thickness of the specimen and “T” is the height of the specimen. The results were also recorded in MPa. The specimens that broke into more than two pieces were excluded from the study. In this study CS and DTS were measured by a specialist in material engineering, who was blind to the process of preparing the specimens. Data was analyzed by Student’s t-test using SPSS 15 software.

## Results

The comparison of the CS in type III and type IV stone casts for irradiated and un-irradiated specimens are presented in Table 1. According to the results of Student’s t-test, microwave irradiation had no effect on the CS of stone casts of both types.

**Table 1:** The results of Student’s t-test for compressive strength (mean ± SD) of type III and type IV stone casts irradiated by microwave compared with un-irradiated casts

Stone type	Sample number	Radiation	Compressive strength (MPa)	P-value
III	10	No	26.31±1.54	P>0.05
III	10	Yes	28.1±1.39	P>0.05
IV	10	No	38.66±2.23	P>0.05
IV	10	Yes	40.41±1.65	P>0 .05

The comparison of DTS in type III and type IV stone casts for irradiated and un-irradiated casts are presented in Table 2. The results indicated an increase in the DTS of the two stone types following irradiation.

**Table 2:** Student’s t-test results for diametral tensile strength (mean ± SD) of type III and type IV stone casts irradiated by microwave compared with un-irradiated casts

Stone type	Sample number	Radiation	Diametral tensile strength (MPa)	P-value
III	10	NO	3.97±0.59	P<0.01*
III	10	YES	5.23±0.64	P<0.01*
IV	10	NO	5.83±0.69	P<0.01*
IV	10	YES	8.71±0.94	P< 0.01*

* Statistically significant

## Discussion

The aim of this study was to evaluate the effect of microwave disinfection on DTS and CS of type III and IV dental stones. The results showed that the DTS of both types in the group irradiated with a 600-W beam for 3 minutes was more than that in the groups that did not undergo irradiation; however, there were no differences in the CS.

The results of this study are consistent with those of previous studies which have shown that the DTS in stone casts dried by microwaves with an energy level of 600 W for 10 minutes was more than that in air-dried casts 2 hours after irradiation.^22^ An increase in DTS in type III and IV stone casts after irradiation with an energy level of 900 W for 5 minutes was also observed later.^17^

Lower energy levels have been suggested for the process of irradiating and heating of gypsum products, especially in denser casts, in order to prevent crack formation as a result of rapid formation and release of vapor.^18^ Although casts dried with microwave with maximum power for five minutes had higher surface hardness compared to air-dried casts after 4 hours, an ultimate decrease in surface hardness was observed after 24 hours.^18^ According to another study, an energy level of 550 W for about five minutes increases CS in type III stone casts after 24 hours.^20^ However, another study showed increases in CS in type III and IV stone casts at an energy level of 900 W for about 5 minutes as mentioned above.^17^

The controversial results might be attributed to different techniques of irradiation applied; when the irradiation period at 900 W is divided into two 2.5-minute periods, the interruption in the period can decrease the temperature and prevent damage from high levels of energy.

One of the main reasons for lower strength of oven-dried casts compared to air-dried ones is lack of water molecules in the early hours, which are necessary to form, grow, sediment and join together the crystals of CaSO_4_.2H_2_O under energetic microwaves, resulting in dehydrated poor casts.^20,21,24,25^

Additionally, in order to achieve maximum strength, the excess water of stone casts should be removed, a process which takes seven days after pouring in the ambient temperature.^25^ The comparison of the strength of the specimens at this stage is useful in detecting even minor effects of microwave irradiation on casts. This fact was one of the considerations in the current study. Another consideration in the present study was the seven-time irradiation as an average number of times that casts need to be disinfected during prosthodontic procedures.^19,6^ Moreover, wetting the casts before irradiation was an attempt to imitate the condition of casts in microbiologic studies.^23^

Studying the effects of microwave irradiation on the physical properties, such as surface roughness, detail reproduction and dimensional stability; mechanical properties such as surface hardness and abrasion resistance, and the research for techniques that increase microbicidal efficiency of microwave can be new fields of investigation.

## Conclusion

Under the limitations of this study, microwave irradiation of type III and IV stone casts at 600-W energy level for 3 minutes could enhance their DTS without affecting their CS.
